# Highly Efficient Synergistic Chemotherapy and Magnetic Resonance Imaging for Targeted Ovarian Cancer Therapy Using Hyaluronic Acid‐Coated Coordination Polymer Nanoparticles

**DOI:** 10.1002/advs.202309464

**Published:** 2024-09-17

**Authors:** Guang Li, Shengying Shi, Jingxiu Tan, Lijuan He, Qiwen Liu, Feng Fang, Huijiao Fu, Min Zhong, Ziyi Mai, Rui Sun, Kun Liu, Zhenzhen Feng, Peiqin Liang, Zhiqiang Yu, Xuefeng Wang

**Affiliations:** ^1^ Department of Obstetrics and Gynecology The Third Affiliated Hospital Southern Medical University Guangzhou 510630 China; ^2^ Department of Gynecological Oncology and Cervical Lesions Hunan Provincial Maternal and Child Health Care Hospital Changsha 410013 China; ^3^ Department of Nursing Nanfang Hospital Southern Medical University Guangzhou 510000 China; ^4^ Department of Pharmacy The First Affiliated Hospital of Shenzhen University Shenzhen Second People's Hospital Shenzhen 518035 China; ^5^ Department of Laboratory Medicine Dongguan Institute of Clinical Cancer Research Affiliated Dongguan Hospital Southern Medical University Dongguan 523018 China; ^6^ School of Pharmacy Guangdong Medical University Dongguan 523808 China

**Keywords:** ferroptosis, gsh depletion, integrated diagnosis and treatment, nuclear magnetic resonance imaging (MRI), ovarian cancer (OC)

## Abstract

The diagnosis and treatment of ovarian cancer (OC) are still a grand challenge, more than 70% of patients are diagnosed at an advanced stage with a dismal prognosis. Magnetic resonance imaging (MRI) has shown superior results to other examinations in preoperative assessment, while cisplatin‐based chemotherapy is the first‐line treatment for OC. However, few previous studies have brought together the two rapidly expanding fields. Here a technique is presented using cisplatin prodrug (Pt‐COOH), Fe^3+^, and natural polyphenols (Gossypol) to construct the nanoparticles (HA@PFG NPs) that have a stable structure, controllable drug release behavior, and high drug loading capacity. The acidic pH values in tumor sites facilitate the release of Fe^3+^, Pt‐COOH, and Gossypol from HA@PFG NPs. Pt‐COOH with GSH consumption and cisplatin‐based chemotherapy plus Gossypol with pro‐apoptotic effects displays a synergistic effect for killing tumor cells. Furthermore, the release of Fe^3+^ at the tumor sites promotes ferroptosis and enables MRI imaging of OC. In the patient‐derived tumor xenograft (PDX) model, HA@PFG NPs alleviate the tumor activity. RNA sequencing analysis reveals that HA@PFG NPs ameliorate OC symptoms mainly through IL‐6 signal pathways. This work combines MRI imaging with cisplatin‐based chemotherapy, which holds great promise for OC diagnosis and synergistic therapy.

## Graphical abstract

The diagnosis and treatment of ovarian cancer (OC) are still a grand challenge, magnetic resonance imaging (MRI) is a typical diagnostic method for accurately diagnosing OC, while cisplatin is the first‐line treatment method. Here, the study reports nanoparticles (HA@PFG NPs) composed of Fe^3+^, Pt‐COOH, and Gossypol that integrate MRI with cisplatin‐based chemotherapy.

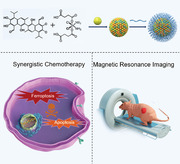

## 1 Introduction

Ovarian cancer (OC) is a silent disease with an extremely poor prognosis, more than 70% of OC cases are already at an advanced stage with a 5‐year overall survival (OS) rate less than 30% when they are diagnosed.^[^ ^1^, ^2^ ^]^ The high rate of mortality and relatively low rate of survival are mainly due to the lack of methods for early diagnosis and effective therapeutic intervention.^[^ ^3^ ^]^ Early detection and diagnosis of OC could significantly improve patient outcomes.^[^ ^4^, ^5^ ^]^ Ultrasound, CT, and MRI are commonly used for preoperative diagnosis of OC.^[^ ^6^ ^]^ Among these imaging techniques, MRI is currently one of the most powerful diagnostic tools with high spatial resolution and soft tissue contrast, which was widely used in diagnosis, particularly differential diagnosis, and preoperative therapeutic options.^[^ ^7^ ^]^ Gadolinium‐based contrast agents are widely used in clinical MRI. However, gadolinium‐based contrast agents have been associated with nephrogenic systemic fibrosis in patients with renal insufficiency.^[^ ^8^, ^9^, ^10^ ^]^ Therefore, the development of new contrast agents is a very urgent need. Fe^3+^ is not only an essential element for living organisms but also a paramagnetic species which can consequently be measured non‐invasively using MRI.^[^ ^11^, ^12^, ^13^ ^]^ This makes Fe^3+^ a promising candidate as a safe contrast agent.

Cisplatin‐based chemotherapy is the first‐line treatment for OC.^[^ ^14^, ^15^ ^]^ Various forms of nanomedicine based on cisplatin have been widely studied.^[^ ^16^, ^17^, ^18^, ^19^ ^]^ However, their application is always limited by their low Pt‐loading capacity, which increased dosage of cisplatin and caused side effects such as nephrotoxicity. The emergence of metal‐based nanoparticles (NPs) provides new opportunities to solve this problem. Metal‐based NPs, connected by coordination bonds between transition metal ions and chemotherapeutics, have become a hotspot in the field of nanomedicine.^[^ ^20^, ^21^, ^22^, ^23^ ^]^ The no‐carrier NPs formed by coordination bonds exhibit high drug loading capacity, convenient adjustment of drug ratio, and good responsiveness to the tumor microenvironment.^[^ ^24^, ^25^, ^26^, ^27^ ^]^ Recently, such NPs have attracted extensive attention in bioimaging, drug delivery, and surface coating due to their excellent physicochemical properties, offering a superior approach to inhibition of tumor growth.^[^ ^28^, ^29^, ^30^, ^31^, ^32^, ^33^, ^34^ ^]^ There are few studies on the combination of cisplatin prodrug and metal polyphenol NPs.

Gossypol, a natural compound extracted from cotton plants, exerts anti‐tumor effects by strong binding affinity to apoptosis‐associated proteins, such as Bcl‐2, Bcl‐xL, and Mcl‐1.^[^ ^35^ ^]^ In addition, the symmetrical structure of gossypol provides coordination sites with metals and other phenolic structures. These characteristics make it a good candidate for cisplatin‐based synergistic chemotherapy.^[^ ^36^ ^]^

**Scheme 1:**
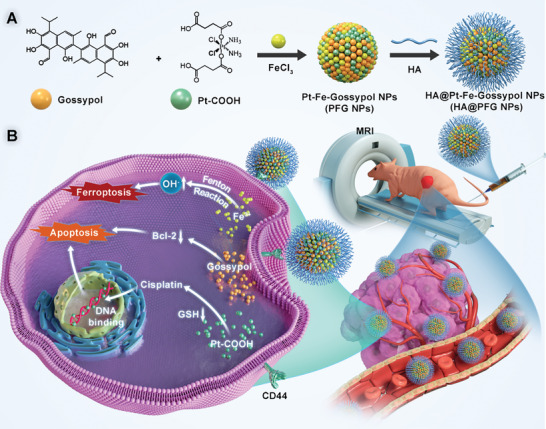
A) The synthetic procedure for HA@PFG NPs and B) schematic illustration of HA@PFG NPs for MR imaging and chemotherapy.

Based on above demands, we have therefore developed a complex nanocarrier based on metal coordination bonds formed between gossypol, Pt‐COOH (cis, cis, trans‐Pt‐(NH_3_)_2_Cl_2_(OOCCH_2_CH_2_COOH)_2_) and Fe^3+^ (**Scheme** **1**). After coating with hyaluronic acid (HA), the HA@PFG NPs exhibited specific targeting behavior, strong‐penetrability, controllable drug release properties, and good contrast imaging capabilities both in vitro and in vivo. Mechanistically, cisplatin‐based chemotherapy, ferroptosis mediated by Fe^3+^, and redox imbalance play important roles in anti‐tumor processes. These characteristics indicate that HA@PFG NPs are excellent nanocarriers with good potential for clinical application, integrating diagnosis and targeted tumor treatment.

## 2 Results and Discussion

### 2.1 Synthesis and Characterization of HA@PFG NPs

**Figure 1:**
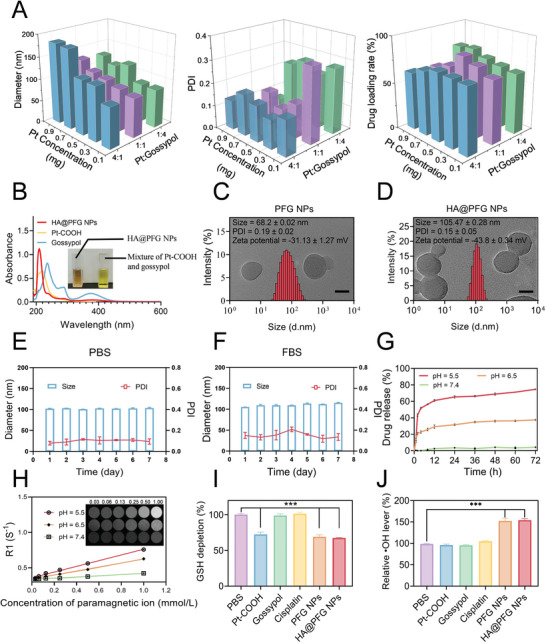
Characterization of HA@PFG NPs. A) Changes in average diameter, PDI and drug loading rate of the HA@PFG NPs by adjusting the Pt‐COOH and Gossypol ratio. The mass of Pt‐COOH was fixed to be 5 mg during the preparations; B) UV–vis spectra of Pt‐COOH, Gossypol and HA@PFG NPs; TEM images of PFG NPs (C) and HA@PFG NPs (D). Scale bar: 50 nm; Stability of PFG NPs and HA@PFG NPs in PBS (E) and 10% fetal bovine serum (F) over 7 days; G) Drug released from HA@PFG NPs at different pH value; H) The T1‐weighted MRI and relaxation rate of HA@PFG NPs at different pH value; Ferroptosis of different groups evaluated by GSH (I) and ⋅OH (J) levels. *P < 0.05, **P < 0.01, and ***P < 0.001; ns indicates *P* > 0.05.

The Pt(IV) prodrug Pt‐COOH was prepared in a two‐step process as illustrated in Figure S1 (Supporting Information). The synthesis method for the reference HA@PFG NPs was also provided in support information. Interestingly, the size and polydispersity index (PDI) of the obtained HA@PFG NPs could be fine‐tuned by simply varying the Pt‐COOH/gossypol ratio and Pt concentration (**Figure** **1**A). The size of HA@PFG NPs increased as the concentration of Pt was increased due to the intense interaction between Pt‐COOH and gossypol. When the Pt‐COOH/gossypol molar ratio was 1:1, Pt‐COOH coordinated gossypol to form optimal NPs with a diameter of 105.47 ± 0.28 nm and PDI of 0.15 ± 0.05 (Figure 1A,D). As shown in Figure S4 (Supporting Information), the Tyndall effect of PFG NPs and HA@PFG NPs indicated that these NPs were successfully synthesized. Zeta potential changes and color changes could be observed in the synthesis procedure of the HA@PFG NPs (Figures S5 and S6, Supporting Information). Elemental mapping detected by STEM showed that the elements of C, N, O, Pt, and Fe were uniformly distributed in HA@PFG NPs (Figure S7, Supporting Information). The coordination interactions between Fe^3+^, Pt‐COOH, and gossypol were also confirmed by an obvious shift in the UV‐vis spectrum, HA@PFG NPs have a UV absorption peak at 210 nm, and the waveform is completely different from gossypol and Pt‐COOH, confirming that HA@PFG NPs are not a simple mixture of Pt‐COOH and gossypol, but a substance with a new structure has been formed through some interaction force (Figure 1B).

No significant changes in size or PDI of HA@PFG NPs were observed in PBS buffer and 10% FBS over the course of 7 days (Figure 1E,F), which confirmed the stability of the NPs. Since metal coordination bonds are stronger than hydrogen bonds in general,^[^ ^37^ ^]^ the NPs were relatively stable at pH 7.4, with less than 10% of gossypol released after 72 h incubation (Figure 1G). The release rate dramatically increased under acidic conditions, however, with ≈80% of gossypol released over the same experimental period. This is because cleavage of the coordination bonds is sensitive to variations in external pH.^[^ ^38^ ^]^ Therefore, HA@PFG NPs are generally stable in the bloodstream and tissue fluid, avoiding premature drug release and ensuring long half‐life in circulation. Upon reaching the tumor site, the small molecule is rapidly released in the acidic tumor microenvironment.

After the pH‐sensitive linkages are broken at the tumor site, components in the NPs are released. First, the release of Fe^3+^ from HA@PFG NPs enhanced the T1‐weighted MRI signal. T1 images of HA@PFG NPs in solutions at different pH values were scanned by MRI. As shown in Figure 1H, HA@PFG NPs at low pH values led to considerably enhanced T1‐weighted intensity, which indicated the potential for in vivo T1‐weighted MRI. Second, the released Fe^3+^ and Pt‐COOH perturbed the intracellular redox balance via the Fenton reaction, which consumed GSH and generated hydroxyl radicals (Figure 1I,J).

### 2.2 Targeting and Penetration Abilities of HA@PFG NPs

**Figure 2:**
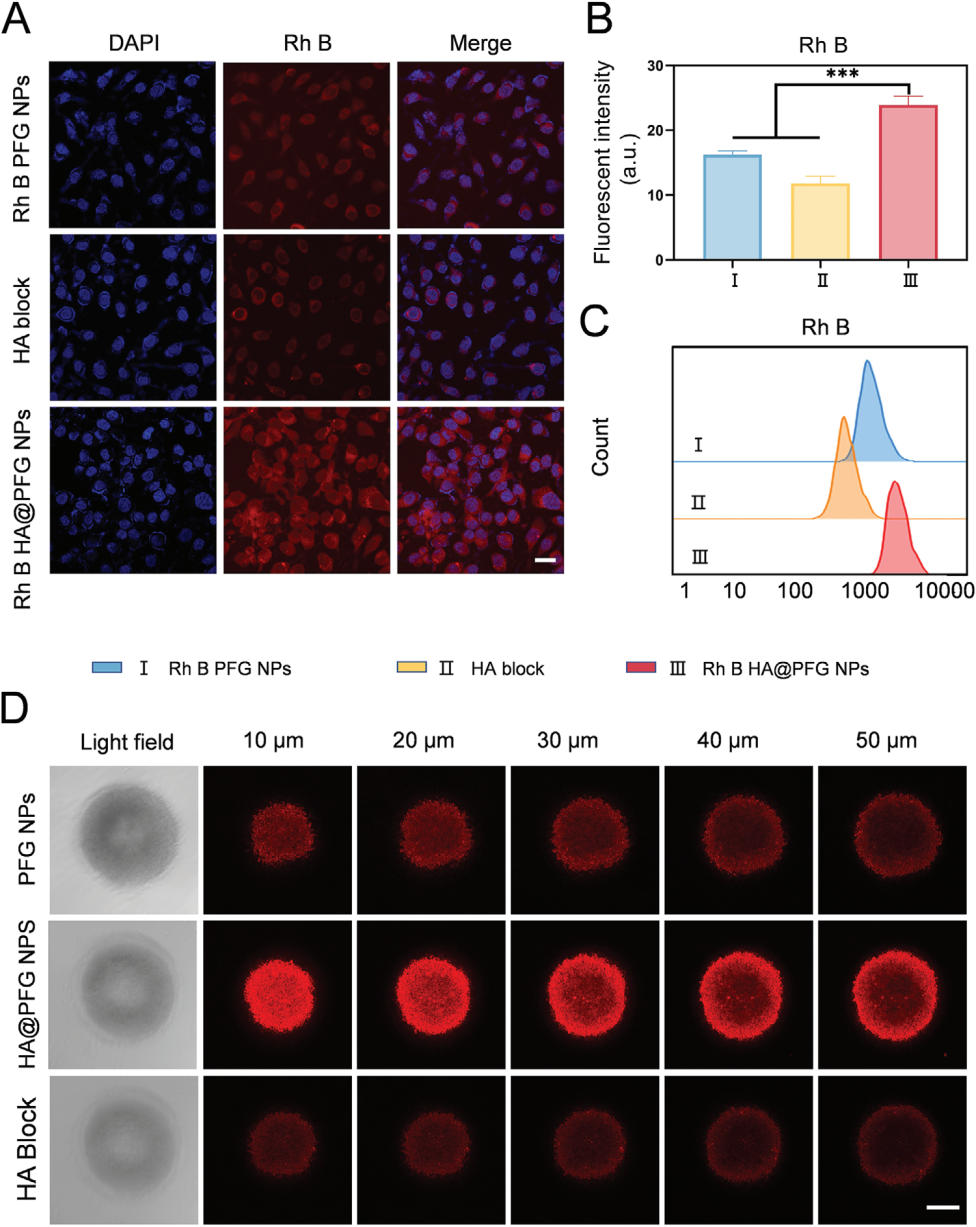
Cell uptake and in vitro permeability of HA@PFG NPs. A) CLSM images of A2780 cells after treating with Rh B labeled NPs for 6h. Scale bar: 25 µm; B) Semi‐quantitative analysis of mean Rho B intensity of intracellular NPs; C) Quantitative analysis of intracellular NPs by flow cytometry; D) CLSM imaging of the 3D sphere penetration at different depths after treatment with NPs for 6h. scale bar: 100 µm.

Targeting ligands play a crucial role in the targeted delivery of NPs to tumor sites.^[^ ^39^ ^]^ The CD44 cell adhesion receptor, which is overexpressed in many types of cancer cells, has frequently been exploited to target cancer cells as the HA receptor.^[^ ^25^, ^29^, ^40^ ^]^ In this study, HA‐coated NPs HA@PFG NPs were synthesized to enhance tumor targeting. As shown in **Figure** **2**A,B, NPs coated with HA significantly increased internalization by A2780 cells, thus emitting the strongest red fluorescence. In striking contrast, when CD44 was blocked or not targeted by HA, the internalization of NPs was reduced and cells exhibited only faint red fluorescence. Similar results were obtained using quantitative flow cytometry (Figure 2C). In addition, after comparing the internalization of NPs at different time points, we found that HA@PFG NPs were internalized in a time dependent manner. Uptake peaked 6 h post‐induction and decreased substantially thereafter (Figure S9, Supporting Information). Besides, CLSM and flow cytometry also confirmed that the cellular uptake of HA@PFG NPs was also significantly higher in tumor cells than in normal cells (Figure S10, Supporting Information).

3D tumor spheroids accurately mimic what is occurring physiologically in the tumor microenvironment, including nutritional deficiencies and hypoxia in the core regions of tumors.^[^ ^41^ ^]^ The high activity of hyaluronidase at the tumor site results in cleavage of the outer HA shell to release the small diameter drugs, contributing to deep penetration of the tumor.^[^ ^42^ ^]^ As shown in Figure 2D and S11 (Supporting Information), compared with PFG NPs, HA@PFG NPs exhibited superior penetration at all scanning depths. More HA@PFG NPs accumulated in 3D tumor spheroids as a result of active targeting and the smaller diameter after degradation.

### 2.3 In Vitro Cytotoxicity and Mechanisms

**Figure 3:**
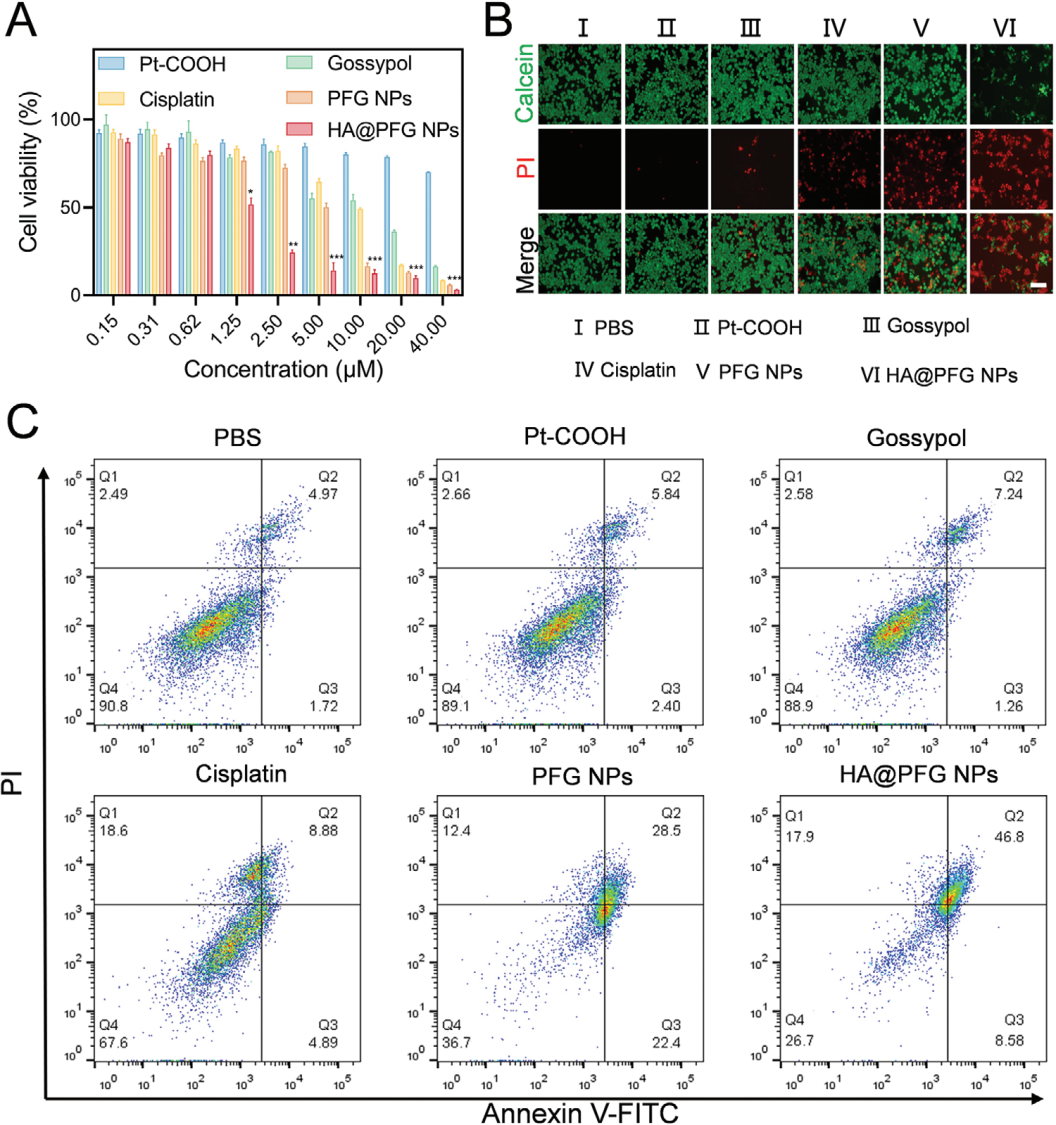
The cytotoxicity of HA@PFG NPs. A) Relative cell viability of A2780 cells after treatment with Pt‐COOH, Gossypol, Cisplatin, PFG NPs, and HA@PFG NPs for 72 h, *P < 0.05, **P < 0.01, and ***P < 0.001; B) Live cells stained with calcein (green) and dead cell nuclei stained with PI (red), Scale bar: 100 µm; C) A2780 cell apoptosis images after different treatments for 24 h.

Inspired by the idea that enhanced uptake of NPs can enhance cytotoxic responses to chemotherapy, the antitumor effect of HA@PFG NPs was further investigated in A2780 cells. As shown in **Figure** **3**A and S12 (Supporting Information), the IC_50_ of cisplatin was 11.56 ± 1.42 µM. The IC_50_ of PFG NPs was 5.48 ± 2.81 µM, and that of HA@PFG NPs was 1.31 ± 0.37 µM. Subsequent experiments confirmed that after 48 h of treatment, the percentage of apoptotic cells in the PBS group was only 6.69%, in the gossypol group was 8.50%, in the cisplatin group was 13.77%, and in the Pt‐COOH group was 8.24%. The percentage of apoptotic cells in the PFG NPs group reached 50.90%. For HA@PFG NPs, the proportion of apoptotic cells reached 55.38% (Figure 3C). Further live/dead experiments also showed that compared with the other groups, HA@PFG NPs resulted in a large amount of red fluorescence, indicating a significant increase in dead cells during this period. HA@PFG NPs were strongly toxic toward A2780 cells, which was consistent with the MTT and apoptosis results (Figure 3B).

**Figure 4:**
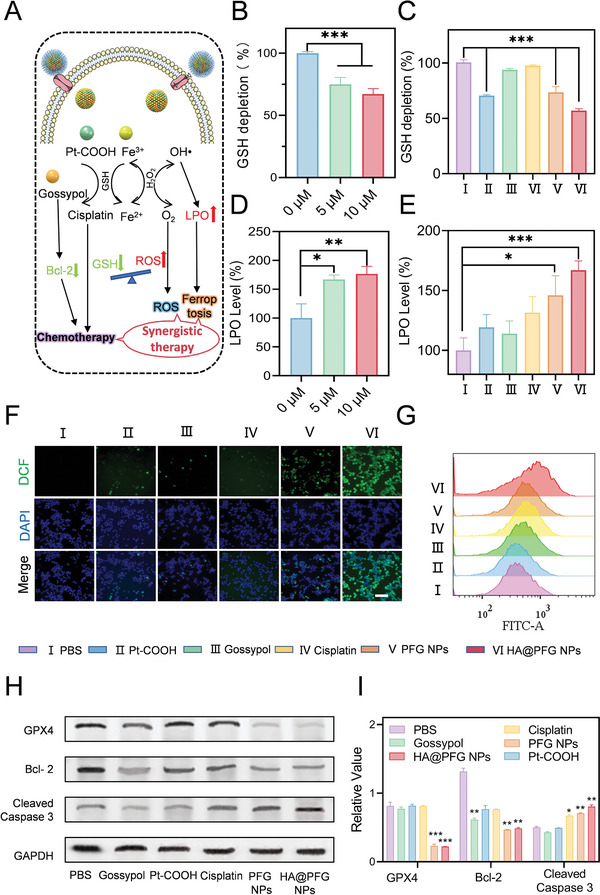
The mechanism of cytotoxicity induced by HA@PFG NPs. A) Schematic illustration of the synergistic mechanism of chemotherapy and ferroptosis; B) Intracellular GSH levels in A2780 cells treated with HA@PFG NPs at different concentrations; C) Intracellular GSH levels in A2780 cells following treatment with different formulations; D) LPO levels in A2780 cells incubated with different HA@PFG NPs concentration; E) LPO levels in A2780 cells following treatment with different formulations; (F) ROS intensity in A2780 cells treated with HA@PFG NPs at different time points was examined by CLSM observation (F) and flow cytometer (G), scale bar: 100 µm; Western blot analysis (H) and quantification (I) of proteins related to ferroptosis and apoptosis in A2780 cells after various treatments. *P < 0.05, **P < 0.01, and ***P < 0.001.

Encouraged by the in vitro antitumor efficacy of HA@PFG NPs, we further interrogated the possible mechanisms of action. The schematic in **Figure** **4**A describes the proposed cytotoxic mechanisms in the synergistic therapeutic effects of HA@PFG NPs. After intracellular uptake, the HA@PFG NPs NPs release remarkable quantities of Fe^3+^, Pt‐COOH, and free gossypol under acidic conditions. Under the joint action of Fe^3+^ and Pt‐COOH, large amounts of the endogenous reducing agent, GSH, are consumed, which perturbs the redox balance and induces ferroptosis. As shown in Figure 4B, a concentration‐dependent decrease of GSH levels was observed in A2780 cells. In addition, intracellular GSH levels in the HA@PFG NPs group were significantly reduced compared with each of the other groups, demonstrating a superior depletion of GSH by the NPs (Figure 4C). In contrast, intracellular lipid peroxide (LPO) levels exhibited an opposite trend (Figure 4D,E). Furthermore, much stronger green fluorescence appeared in A2780 cells treated with HA@PFG NPs compared with other treatments, suggesting enhanced ROS generation (Figure 4F,G). GSH depletion triggered by Fe^3+^ and ROS‐mediated lipid peroxidation are the two essential characteristics of ferroptosis.^[^ ^22^ ^]^ These results support ferroptosis as a main mechanism for HA@PFG NPs cytotoxicity toward cancer cells.

To further explore the underlying molecular mechanisms of HA@PFG NPs cytotoxicity, we assessed relevant functional proteins by western blot. As shown in Figure 4H,I, GPX4 expression was substantially down‐regulated in the presence of Fe^3+^ (PFG NPs and HA@PFG NPs) compared with PBS. In addition, decreased expression was observed in A2780 cells after treatment with HA@PFG NPs, since gossypol acts as a Bcl‐2 inhibitor.^[^ ^35^, ^43^ ^]^ Exposure of A2780 cells to HA@PFG NPs led to significantly higher levels of apoptosis‐related proteins, such as cleaved caspase‐3. In summary, HA@PFG NPs cause apoptosis through induction of ferroptosis by Fe^3+^ and inhibition of Bcl‐2 by gossypol.

### 2.4 RNA‐Sequencing Analysis

**Figure 5:**
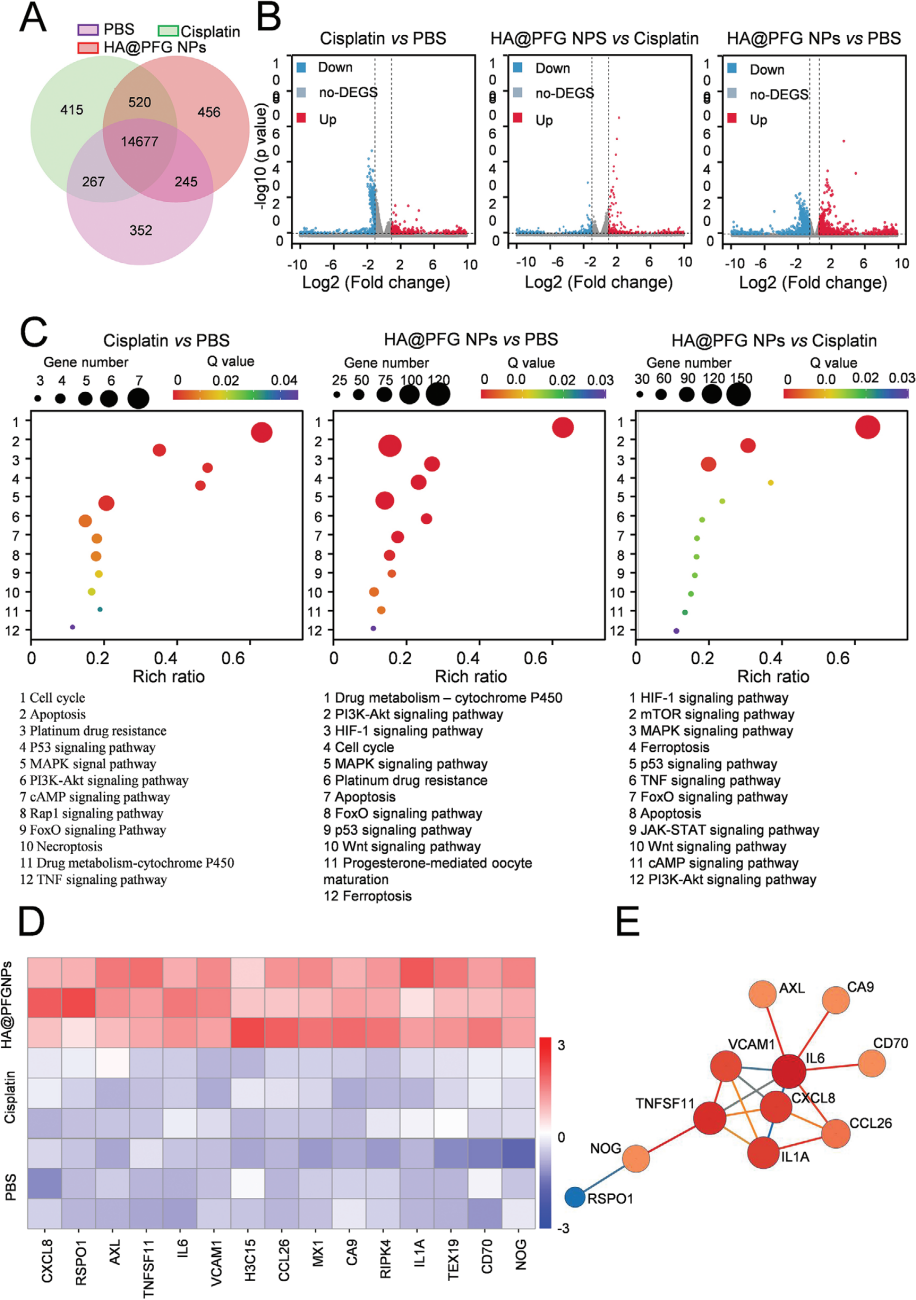
Transcription analysis of A2780 cells treated with cisplatin and HA@PFG NPs by RNA‐sequencing. A) A Venn diagram depicting the number of DEGs in each treatment group; B) Volcano plot of the DEGs using an adjusted p‐value 1.5 as cut off; C) Gene set enrichment analysis plot of Kyoto encyclopedia of genes and genomes (KEGG); D) Heat map of DEGs between each group showing differential gene expression; E) Protein‐protein interaction (PPI) network of DEGs after treatment.

In order to delineate the cytotoxic mechanism of HA@PFG NPs, whole genome RNA sequencing (RNA‐seq) was performed on A2780 cells treated with cisplatin or HA@PFG NPs. A total of 16,582 transcripts were analyzed (**Figure** **5**A). The results showed that, compared with the PBS group, there were 6718 differentially expressed genes (DEGs) in the HA@PFG NPs group, including 3138 up‐regulated (red dots) and 3580 down‐regulated (blue dots). Compared with the cisplatin group, there were 1916 DEGs in the HA@PFG NPs group, including 1008 up‐regulated (red dots) and 908 down‐regulated (blue dots) (Figure 5B). Gene ontology (GO) enrichment analysis revealed that the upregulated genes in the HA@PFG NPs group were associated with biological processes such as HIF‐1, PI3K‐Akt, and MAPK signaling pathways, and ferroptosis (Figure 5C). Inflammation‐related signaling pathways are closely connected with ferroptosis,^[^ ^44^ ^]^ and proinflammatory signals aggravate intracellular oxidative stress and induce excessive lipid peroxidation.^[^ ^45^ ^]^ A large number of inflammation‐related transcription factors (CXCL8, TNFSF11, IL6, VCAM1, and CCL26) were upregulated in the HA@PFG NPs treatment group (Figure 5D), which exacerbated ferroptosis in cancer cells. In particular, IL‐6 plays a key role in iron metabolism and is essential for ferroptosis.^[^ ^46^, ^47^, ^48^, ^49^ ^]^ A unique protein network centered on increased expression of IL‐6 was observed in the HA@PFG NPs group (Figure 5E).

### 2.5 Biodistribution and Magnetic Resonance Imaging (MRI) of HA@PFG NPs In Vivo

**Figure 6:**
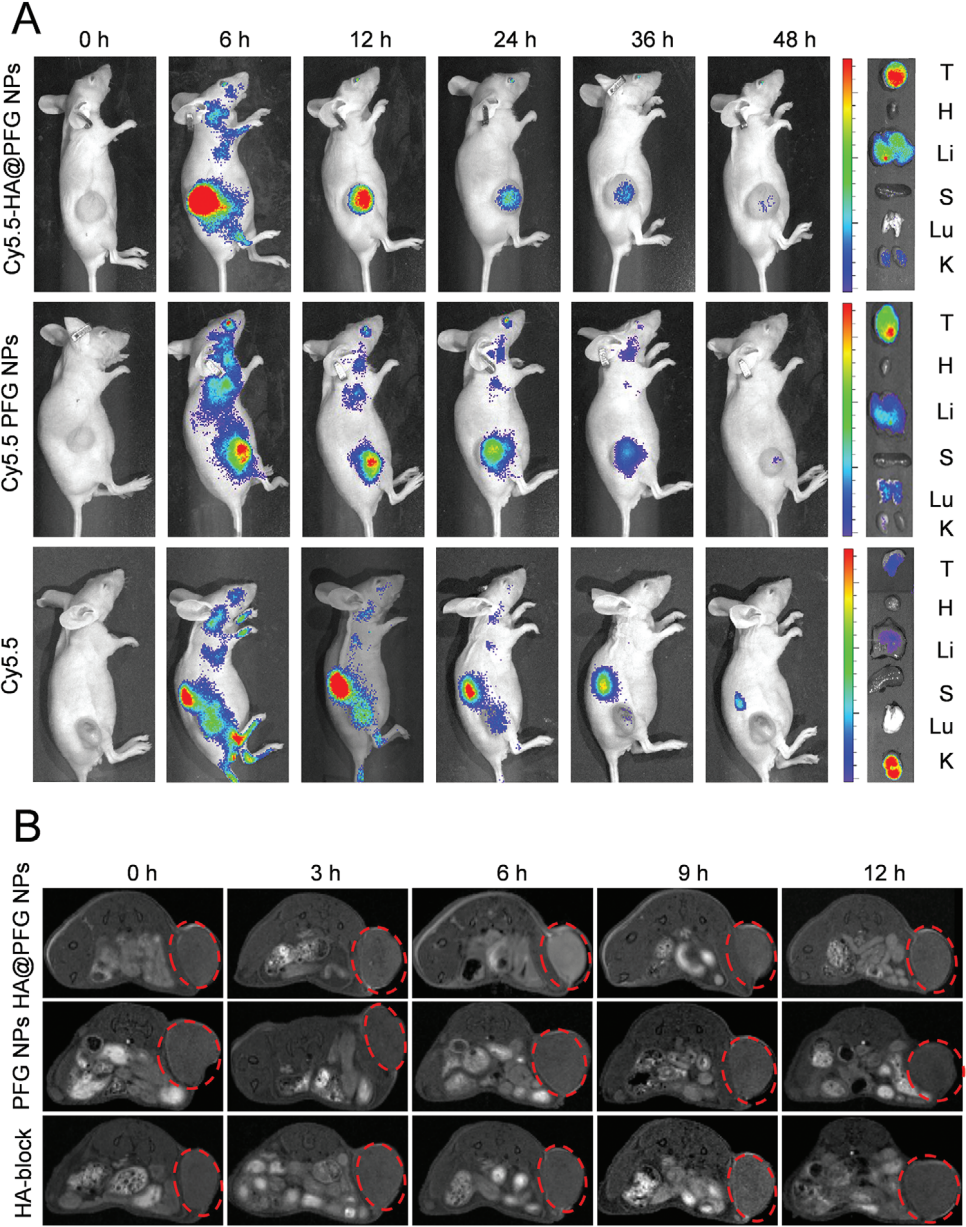
In vivo imaging studies of HA@PFG NPs. A) In vivo and ex vivo fluorescence distribution imaging of Cy5.5 labeled HA@PFG NPs, PFG NPs, and free Cy5.5; B) The T1‐weighted MRI images of PDX tumor‐bearing mice at different time after intravenous injection of various treatments.

To evaluate the biodistribution and tumor accumulation of HA@PFG NPs in vivo, the near‐infrared cyanine dye, Cy5.5, was encapsulated within the hydrophobic core of HA@PFG NPs NPs. As illustrated in **Figure** **6**A, free Cy5.5 was enriched in the kidney and liver. PFG NPs were enriched in tumor, liver, and lung. In stark contrast, the fluorescence signals of the HA@PFG NPs group were relatively stably present at the tumor site. Additionally, the Fe^3+^ contained in HA@PFG NPs confers great potential for MRI.^[^ ^50^ ^]^ A T1‐weighted MRI contrast enhancement was observed in the tumor region 6 h after tail‐vein injection of HA@PFG NPs (Figure 6B and S8, Supporting Information). The in vivo results indicated that HA@PFG NPs accumulated at the tumor site and could be employed for contrast‐enhanced MRI.

### 2.6 Anti‐Tumor Effect of HA@PFG NPs in a PDX Model

**Figure 7:**
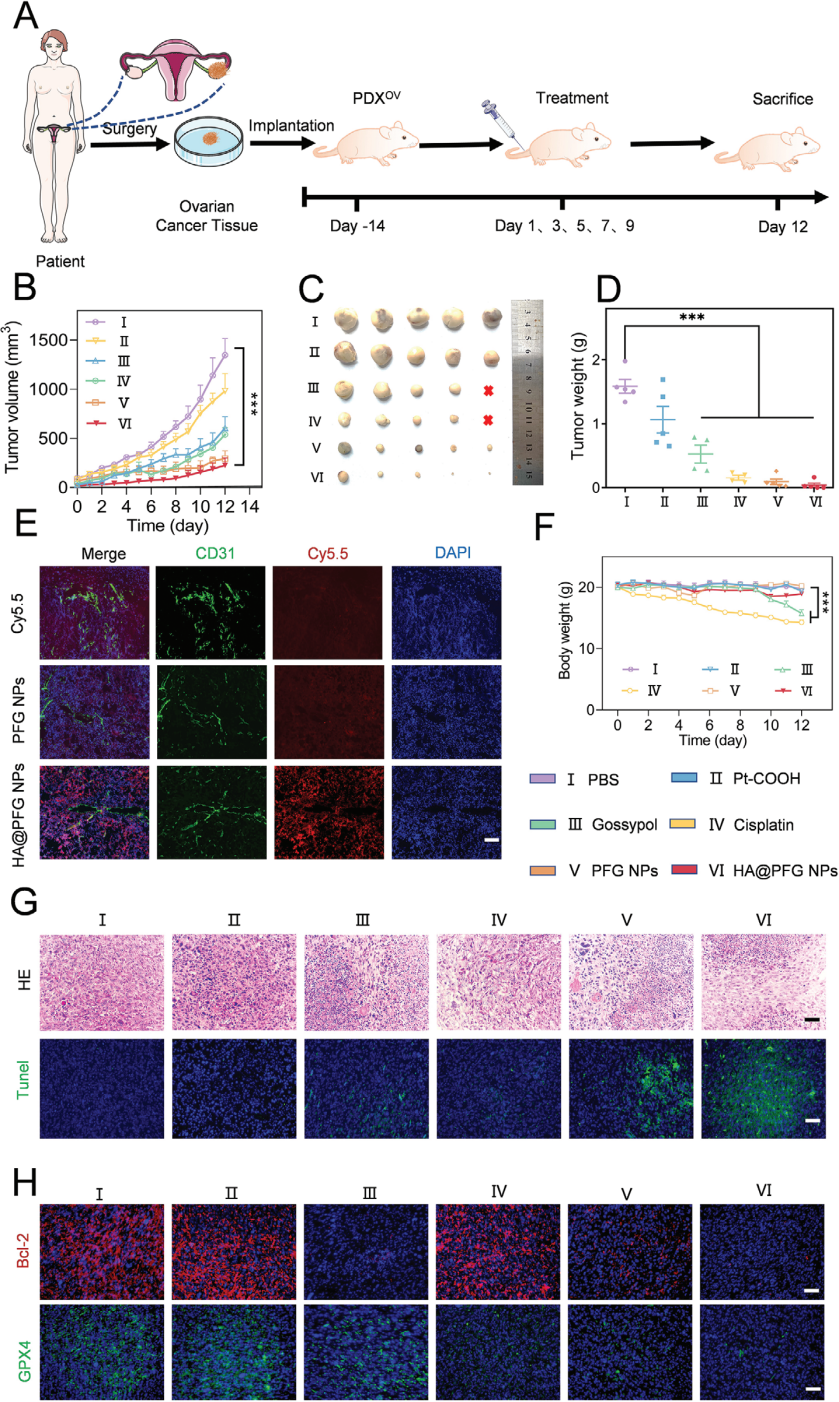
In vitro treatment and permeability ability of HA@PFG NPs. A) The schematic diagram illustrated the PDX model establishment and in vivo treatment process; B) Tumor growth curves, C) photograph, and D) average tumor weights of mice after intravenous injection of different formulations (n = 5); E) Fluorescence intensity of frozen sections of tumor tissues detected by laser confocal microscope, Nuclei were stained with DAPI (blue), the blood vessels were stained with CD‐31 mouse antibodies (green), and the NPs were labeled with Cy5.5 (red). scale bar: 50 µm; F) Body weight of mice during treatment; G) H&E and tunel staining of tumor tissues after treatments. scale bar: 200 µm; H) Immunofluorescence staining of tissues after treatments. scale bar: 200 µm. *P < 0.05, **P < 0.01, and ***P < 0.001; ns indicates *P* > 0.05.

A PDX mouse model was established to evaluate the in vivo therapeutic efficacy of HA@PFG NPs (**Figure** **7**A). Tumor volume and mouse weight were monitored every day, and treatment started when the tumor volume reached 70 mm^3^. The tumor growth curves in different treatment groups are shown in Figure 7B. Tumor volume was 294.33 mm^3^ in the HA@PFG NPs group after 12 days of treatment, while the mean tumor volume in the PBS‐treated group was 1348.04 mm^3^. The tumor weights in the PBS groups were 39.75 times larger (1.59 ± 0.24 *vs*. 0.04 ± 0.07 g) than those in the HA@PFG NPs group (Figure 7C,D). Mice were sacrificed after treatment, and the tumor tissue was collected for hematoxylin−eosin (H&E) staining. Shrinkage of the nucleus and nuclear fragmentation was observed in the HA@PFG NPs group. Finally, a terminal deoxynucleotidyl transferase dUTP nick‐end labeling (TUNEL) assay was carried out on the tumor sections. Consistently, a stronger apoptotic marker was found in HA@PFG NPs‐treated tumor sections (Figure 7G). In addition, the immunofluorescence staining intensity of Bcl‐2 showed that green fluorescence signals in the gossypol, PFG NPs, and HA@PFG NPs groups were much weaker than those of the other groups (Figure 7H and S13, Supporting Information), which could be due to inhibition of Bcl‐2 by gossypol. The WB of tumor tissue also confirmed this result (Figure S14, Supporting Information). GPX4 fluorescence intensity (green) decreased in PFG NPs and HA@PFG NPs treatment groups, which is a main characteristic of ferroptosis (Figure 7H and S15, Supporting Information). These results demonstrate the superior tumor suppressive effects of HA@PFG NPs.

We further investigated the permeability of HA@PFG NPs at the tumor site. Solid tumor tissues were harvested and stained for imaging 6 h after tail vein injections. As presented in Figure 7E, HA@PFG NPs NPs were labeled with fluorescent dye (cy5.5, red), while blood vessels were marked with fluorescent antibodies against CD31 (green). Notably, free cy5.5 and PFG NPs accumulation was confined to perivascular areas of the tumor, while HA@PFG NPs not only accumulated at the perivascular area but were also distributed throughout the tumor sections. This result was attributed to the active targeting role of HA and the small particle size after degradation by hyaluronidase.^[^ ^51^ ^]^

### 2.7 Application Safety Evaluation

The hemolysis test showed that HA@PFG NPs did not cause obvious hemolysis, indicating that the NPs did not interact with the blood components and would not cause harmful effects after being introduced into the blood (Figure S16, Supporting Information). We further investigated the in vivo toxicity of HA@PFG NPs on major organs by serum biochemistry, H&E staining and, body weight measurement. No obvious weight loss (Figure 7F), serum biochemical abnormalities (Figure S17, Supporting Information) or pathological changes (Figure S18, Supporting Information) were observed in pathological sections from HA@PFG NPs‐treated mice, demonstrating the good biosafety of HA@PFG NPs. However, the safety of their clinical application needs to be validated.

## 3 Conclusions

In this work, we developed stable NPs HA@PFG NPs by utilizing the coordination between Fe^3+^, hydroxyl and polyphenols. The NPs were reliably stable under physiological conditions and sensitive to pH at the tumor site. After internalization, HA@PFG NPs exert their cytotoxic effects through synergy of chemotherapy and ferroptosis. Whole‐genome sequencing via RNA‐seq showed that genes involved in HIF‐1, PI3K‐Akt, MAPK and ferroptosis pathways were significantly affected by HA@PFG NPs. In addition, magnetic metal ions (Fe^3+^) were successfully doped into the NPs, endowing HA@PFG NPs with MRI imaging capabilities. Notably, due to the enhanced tumor targeting ability and vascular permeability, HA@PFG NPs exhibited a potent tumor‐suppressive effect in an OC PDX model. Collectively, tumor growth inhibition and MRI enhancement were realized through an integrated diagnosis and treatment system, which facilitates possible clinical transition of HA@PFG NPs.

## 4 Experimental Section

### Materials and Measurements

Cisplatin, hydrogen peroxide (H_2_O_2_) and succinic anhydride were purchased from Energy Chemical (Shanghai, China). HA was sourced from Shanghai Yuanye Bio‐Technology Co., Ltd. (Shanghai, China). The apoptosis detection kit, reactive oxygen species (ROS) assay kit and live/dead staining kit were obtained from Beyotime (Shanghai, China). Lipid peroxidation (LPO) assay kits were purchased from Jiancheng Bioengineering Institute (Nanjing, China). anti‐GPX4, anti‐Bcl‐2, anti‐P53, anti‐γ‐H_2_A.X, and anti‐ GAPDH antibodies were purchased from Abcam (Cambridge, UK), anti‐Cleaved Caspase 3 antibody was purchased from Bioss (Beijing, China). Cell culture vessels and 20 mm glass dishes were purchased from Nest Biotechnology (Wuxi, China).^1^H nuclear magnetic resonance (NMR) spectra were recorded on a 400 MHz NMR spectrometer (Bruker, Karlsruhe, Germany). In vitro MRI was performed using a 7.0 T small animal MRI scanner (Bruker, Karlsruhe, Germany). The stability of HA@PFG NPs under physiological conditions was investigated in phosphate buffered saline (PBS) and 10% fetal bovine serum (FBS, HAKATA, Shanghai, China).

### Cell Culture and Animal Model

A2780 cells were originally obtained from Suncell Biotechnology Co., Ltd. (Wuhan, China). Cells were cultured in RPMI 1640 medium (Sperikon Life Science & Biotechnology co., ltd. China) containing 10% FBS (Inner Mongolia Opcel Biotechnology Co., Ltd. China) and 1% penicillin/streptomycin (Gibco, Grand land, USA) at 37 °C under 5% CO_2_. BALB/c nude mice were purchased from Guangdong Medical Laboratory Animal Center. The OC PDX model was provided by the research group led by Professor Haihua Xiao of the Beijing Institute of Chemistry, Chinese Academy of Sciences. All animal experiments were performed under a protocol approved by the Southern Medical University Animal Laboratory Center (LAEC‐2020‐093).

### Synthesis of Pt‐COOH and HA@PFG NPs

Pt‐COOH was synthesized according to the process illustrated in Scheme 1 (Supporting Information). Briefly, cisplatin (300 mg) was suspended in deionized water and sonicated for 30 min. An aqueous solution of H_2_O_2_ (30 wt.%, 10 mL) was then added dropwise to the solution and the mixture was stirred continuously for 5 h at 70 °C followed by stirring overnight at 4 °C. The product was collected by filtration, washed with ethanol/diethyl ether, and then vacuum‐dried. The collected cis, cis, trans‐Pt(NH_3_)_2_Cl_2_(OH)_2_ complex (237 mg) was reacted with succinic anhydride (140 mg) in dimethyl sulfoxide (DMSO), and the mixture was stirred and heated at 50 °C for 12 h with protection from light. The reaction mixture was concentrated, precipitated in cold diethyl ether and stored at −20 °C for 30 min. Finally, pale yellow cis, cis, trans‐Pt‐(NH_3_)_2_Cl_2_(OOCCH_2_CH_2_COOH)_2_ (Pt‐COOH, 176.27 mg) was collected. The ^1^H NMR spectrum of Pt‐COOH is shown in Figure S2 (Supporting Information).

Gossypol (5.18 mg) and Pt‐COOH (5.34 mg) were dissolved in DMSO (500 µL) and added drop‐wise to a deionized water containing Fe^3+^(2.6mg, 10 mL) (Figure S3, Supporting Information). Tris solution (20mmol, 6.6mL) was then added rapidly and flash mixed. Subsequently, a solution of HA (11 mg) dissolved in deionized water (200 µL) was added. Finally, the resulting solution was purified by dialysis (MW = 3500 Da) and collected (Figure S4, Supporting Information). The loading efficiency and loading content were calculated by the following equations:

(1) $$\mathrm{EE}\left(\mathrm{Encapsulation}\;\mathrm{efficiency}\right)\left(\%\right)=\left(\mathrm{Mloaded}/\mathrm{Madded}\right)*100$$

(2) $$\mathrm{DE}\left(\mathrm{Drug}\;\mathrm{loading}\;\mathrm{efficiency}\right)\left(\%\right)=\left(\mathrm{Mloaded}/\left(\mathrm{Madded}+M\right)\right)*100$$

### Cellular Uptake and Targeting Effect

For cellular uptake experiments, A2780 cells were seeded in 6‐well plates at a density of 5 × 10^5^ cells per well and cultured overnight. After incubation with Rh B (Rhodamine B)‐labeled PFG NPs or Rh B‐labeled HA@PFG NPs for 2, 4, 6, and 8 h, the cells were fixed with 4% paraformaldehyde and the nuclei sequentially stained with 4’,6‐diamidino‐2‐phenylindole (DAPI) for confocal laser scanning microscopy (CLSM) imaging. In addition, treated cells were collected and analyzed by flow cytometry (BD FACS Aria, USA).

For tumor cell targeting analysis, free HA was added to A2780 cells 40 min before the experiment to bind to surface CD44 receptors. Then, after incubation with Rh B‐labeled PFG NPs or Rh B labeled HA@PFG NPs for 6 h, the fluorescence signal was measured by CLSM and flow cytometry as above.

### Three‐Dimensional Multicellular Spheroid Model (A2780)

To evaluate the penetration ability of HA@PFG NPs, a 3D‐tumorsphere model was constructed. A2780 cells were seeded in a 96‐well agarose‐coated plate at a density of 5 × 10^5^ cells per well and incubated for 10 days. The 3D‐tumorspheres that formed were collected and treated with Rh B‐labeled PFG NPs or Rh B‐labeled HA@PFG NPs for 6 h. HA block was performed as described in section 2.5. Penetration depth was scanned from top to middle zone using a laser confocal microscope (Zeiss, Germany).

### Intracellular ROS Detection

A fluorescent probe (DCFH‐DA) (Shanghai Beyotime, Cat#S0033S) was used after different treatments of A2780 cells. Intracellular ROS production was detected by fluorescence microscopy and flow cytometry.

### Detection of Intracellular GSH and LPO

A2780 cells were seeded into 10 cm dishes at density of 5 × 10^6^ cells per dish and cultivated for 24 h. After treatment with PBS, Pt‐COOH, gossypol, cisplatin, PFG NPs, or HA@PFG NPs (Pt: 5 µM; gossypol: 5 µM) for 24 h, cells were collected and freeze–thawed three times with liquid nitrogen. Intracellular levels of GSH were determined using an assay kit according to the manufacturer's instructions (Beijing Solarbio Science & Technology Co., Ltd. Cat# BC1175). Levels of intracellular lipid peroxidation (LPO) were assessed by measuring malondialdehyde (MDA) concentrations. Cell processing was the same as described above. The assay kit was used according to the manufacturer's instructions (Nanjing Jiancheng Bioengineering. Cat# A003‐4‐1).

### In Vitro Cytotoxicity Evaluation

A2780 cells were cultured in 96‐well cell culture plates overnight. Cells were treated with PBS, Pt‐COOH, gossypol, cisplatin, PFG NPs or HA@PFG NPs at Pt and gossypol concentrations of 0.3–40 µM. After incubation for 72 h, 3‐(4,5‐dimethylthiazol‐2‐yl)‐2,5‐diphenyl‐2H‐tetrazolium bromide (MTT) reagent (5 mg/mL) was added and incubated for an additional 4 h. Finally, the absorbance at 490 nm was measured using a microplate reader (Thermo Fisher, USA).

### Western Blot

A2780 cells were seeded on 6‐well plates overnight, the total proteins were collected after various treatments and quantified by a BCA Assay kit. Then, equal amounts of protein were resolved onto a 10% SDS‐PAGE gel and transferred onto PVDF membranes. After blocking with 5% skim milk, the PVDF membranes were incubated with primary antibody at 4 °C overnight. Subsequently, the PVDF membranes were incubated with secondary antibodies at room temperature for 2 h. Finally, the membranes were visualized by an image analysis system (Protein Simple, USA).

### In Vivo Imaging

The in vivo experiment was conducted when the PDX tumor volume reached ≈200 mm^3^. The mice were treated with free Cy5.5 and Cy5.5 labeled HA@PFG NPs via the tail vein. After a predetermined time of 1, 2, 4, 8, 12, or 24 h, NIR images of NP biodistribution were obtained using an IVIS in vivo system (745/820 nm). The tumor‐bearing mice were sacrificed after 24 h. The tumor and other major organ tissues (the heart, liver, spleen, lungs, and kidneys) were harvested, and the fluorescence intensity of IR783 in the organs was assessed after rinsing in PBS.

### In Vivo Tumor Penetration

For tumor penetration analysis, OC PDX model tumor‐bearing mice were injected with free Cy5.5, Cy5.5‐labeled PFG NPs, and Cy5.5‐labeled HA@PFG NPs via the tail vein. Mice were sacrificed 6 h after injection. Tumor tissues were collected and washed three times with PBS, followed by preparation of 6 µm thick sections. The sections were incubated with fluorescein isothiocyanate (FITC)‐labeled anti‐CD31 antibody (green) at 4 °C overnight. Finally, tumor nuclei were labeled with DAPI (blue). The tumor sections were observed by CLSM.

### MRI Phantom Study

Different concentrations (0.03, 0.06, 0.125, 0.25, 0.50, and 1.00 mM of Fe^3+^) of HA@PFG NPs were prepared at pH 7.4 (or pH 5.5 and pH 6.5) for the in vitro MRI phantom study. Longitudinal relaxation times (T1) were measured at 298 K on a Bruker 7.0 T scanner to calculate the relaxation rate.

### In Vivo Antitumor Efficacy

An in vivo patient‐derived xenograft (PDX) model was established to examine the therapeutic effect of HA@PFG NPs. Treatment started when tumor volume reached 70 mm^3^, the body weight and tumor volume of each mouse were monitored daily during treatment. Mice were sacrificed after treatment initiation, blood, tumor tissue and major organs (heart, liver, spleen, kidneys, and lung) were collected for further analysis.

### Statistical Analysis

Data were analyzed using GraphPad Prism 8.0 by ordinary one‐way analysis of variance (ANOVA) and presented as mean ± sd. The significance of differences between two groups was analyzed using Student's t‐test. *P* values less than 0.05 were considered statistically significant (*P < 0.05, **P < 0.01, ***P < 0.001); ns indicates no significant difference.

## Conflict of Interest

The authors declare no conflict of interest.

## Author Contributions

G.L.S.S. and J.T. contributed equally to this work. GL synthesized all the compounds mentioned in this article. GL and SS analyzed and interpreted the data. PL, ZY and XW provided quality control. GL, JT, KL, and ZF conducted cell experiments. GL, LH, QL, FF, HF, MZ, ZM, and RS performed animal experiments. GL wrote the manuscript with input from all co‐authors. ZY supervised the overall research. RS, ZM, and MZ conceived the project, designed the experiments, and revised the manuscript. All authors read and approved the final manuscript.

## Supporting information

Supporting Information

## Data Availability

The data that support the findings of this study are available from the corresponding author upon reasonable request.
