# Supplementary material for: Highly Efficient Synergistic Chemotherapy and Magnetic Resonance Imaging for Targeted Ovarian Cancer Therapy Using Hyaluronic Acid‐Coated Coordination Polymer Nanoparticles
Source: Adv Sci (Weinh). 2024 Sep 17;11(41):2309464. doi: 10.1002/advs.202309464 (PMC11538696; doi:10.1002/advs.202309464)
Supplement: Supplementary file 1 — Supporting Information [file ADVS-11-2309464-s001.docx]

**Support information**


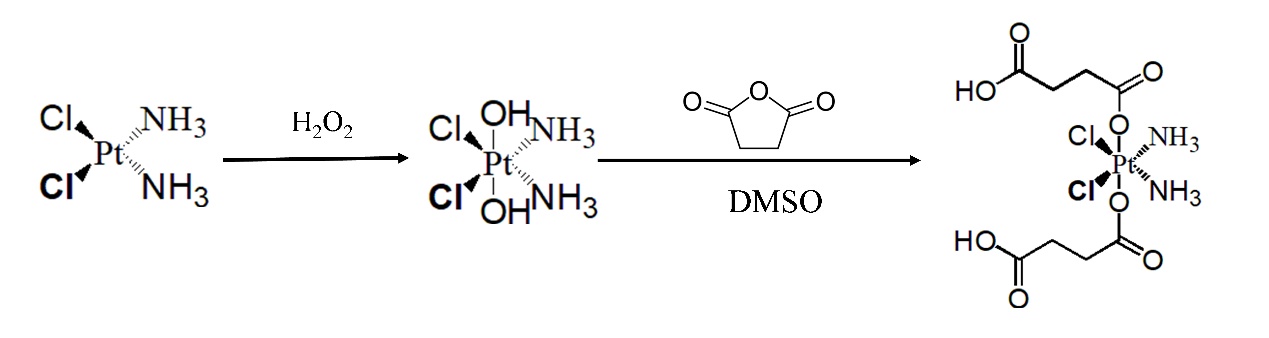


Figure S1. Synthesis route of c,c,t-[Pt(NH_3_)_2_Cl_2_(OOCCH_2_CH_2_COOH)_2_] (Pt-COOH).


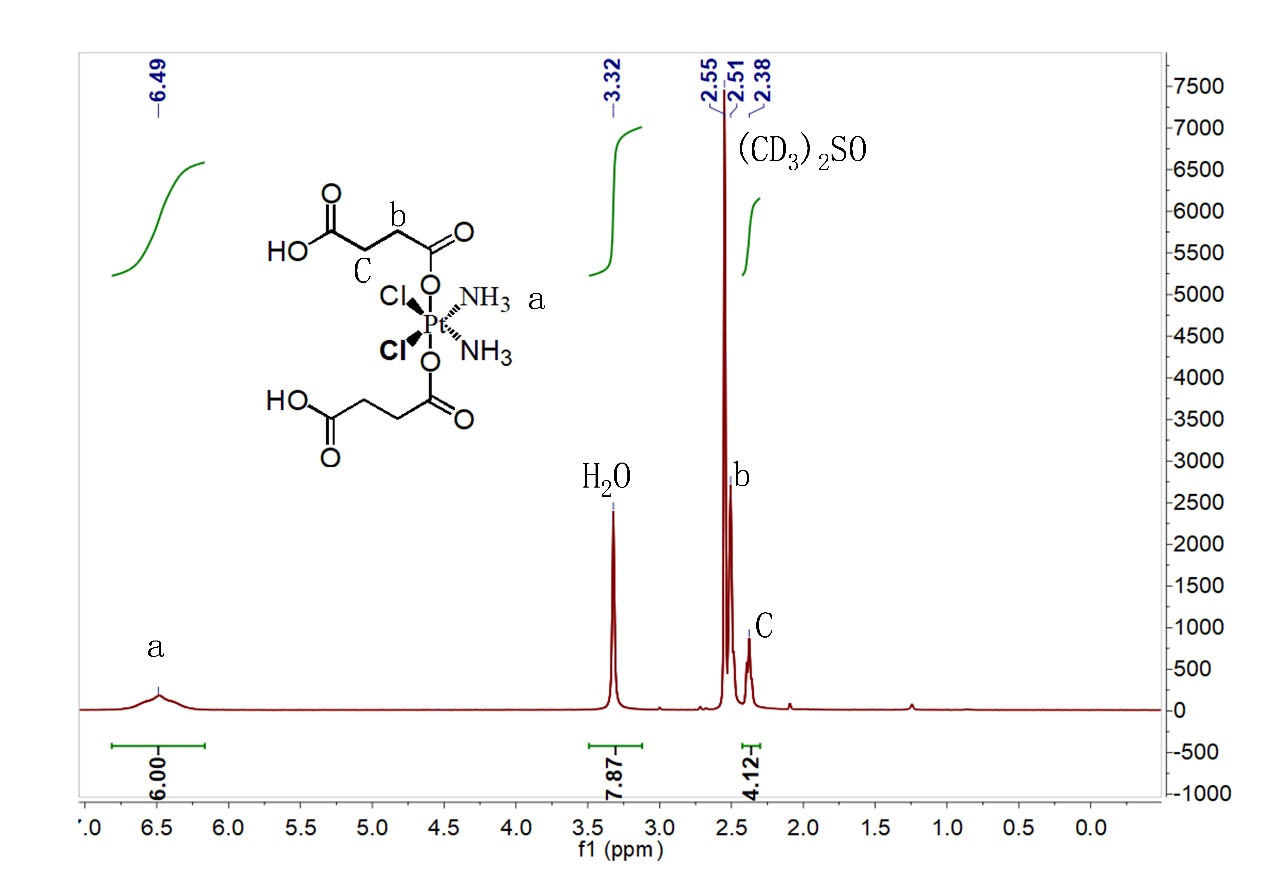


Figure S2. ^1^H NMR spectrum of c,c,t-[Pt(NH_3_)_2_Cl_2_(OOCCH_2_CH_2_COOH)_2_] (Pt-COOH).


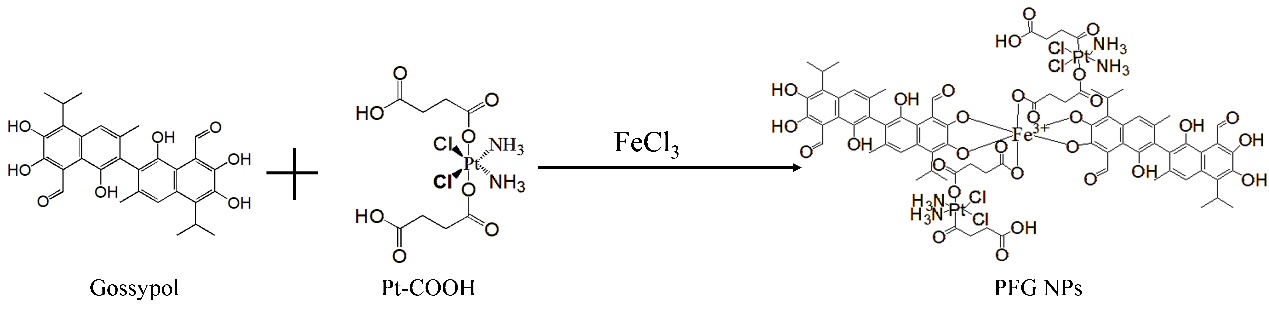


Figure S3. Schematic illustration of the fabrication process of PFG NPs.


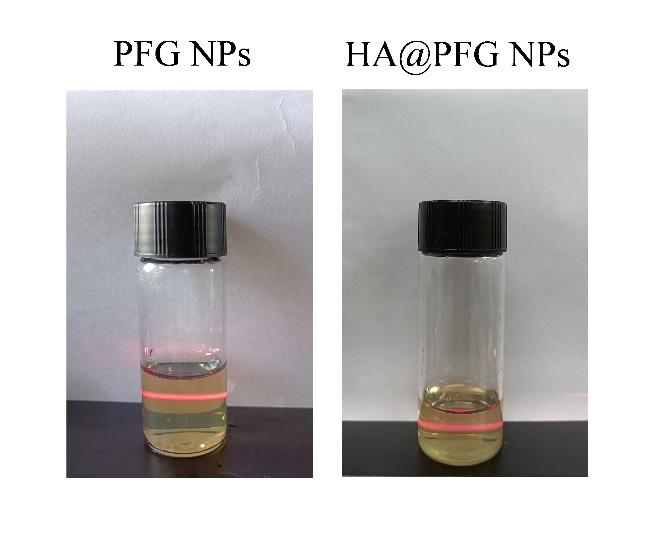


Figure S4. Photostable Image of PFG NPs (left) and HA@PFG NPs (right).


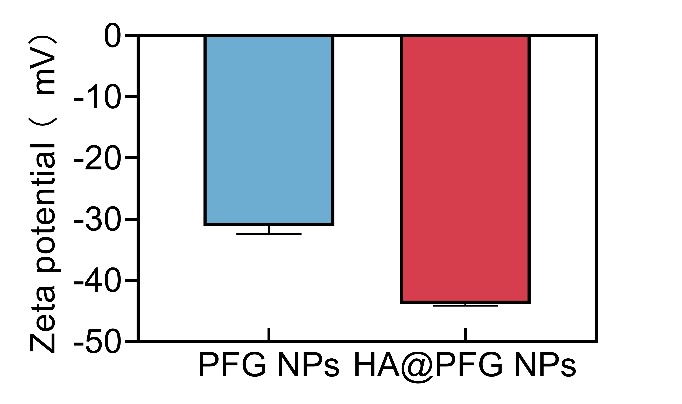


Figure S5. Zeta potential of PFG NPs and hyaluronic acid-coated PFG NPs (HA@PFG NPs).


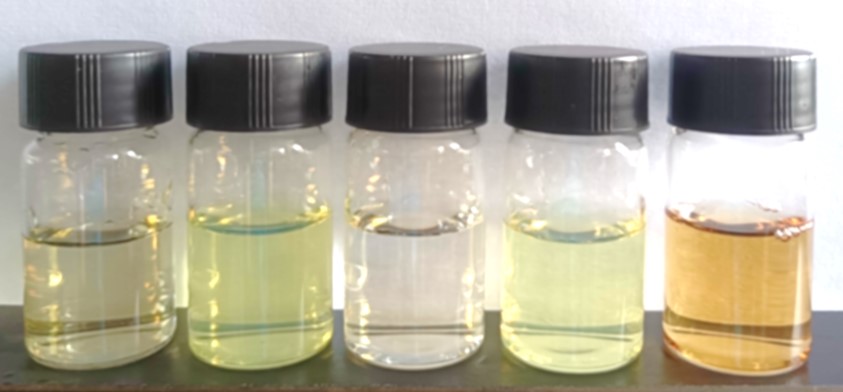


Figure S6. Photograph of FeCl_3_, gossypol, Pt-COOH, gossypol + Pt-COOH, and HA@PFG NPs (from left to right).


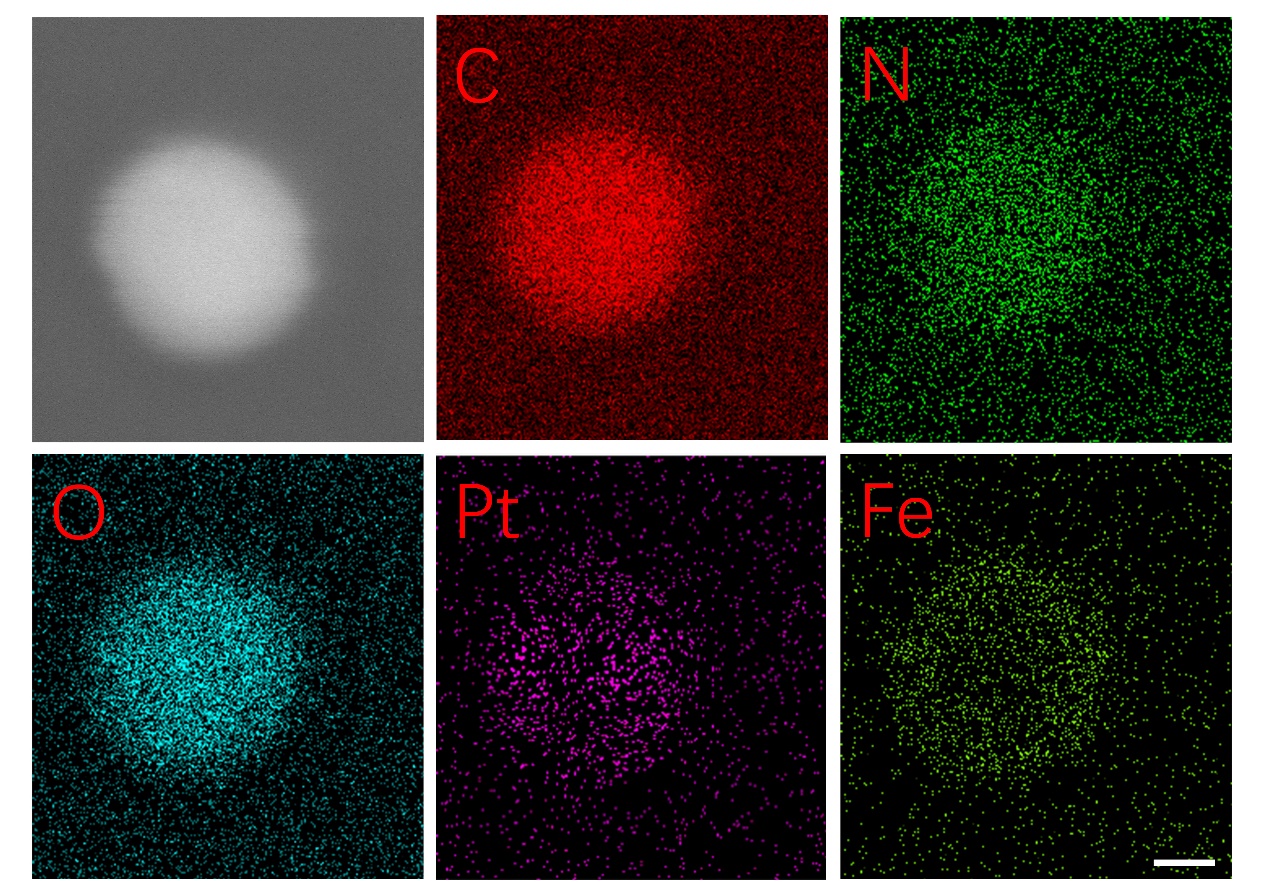


Figure S7. HA@PFG NPs STEM images and corresponding elemental mapping. Scale bar: 50 nm


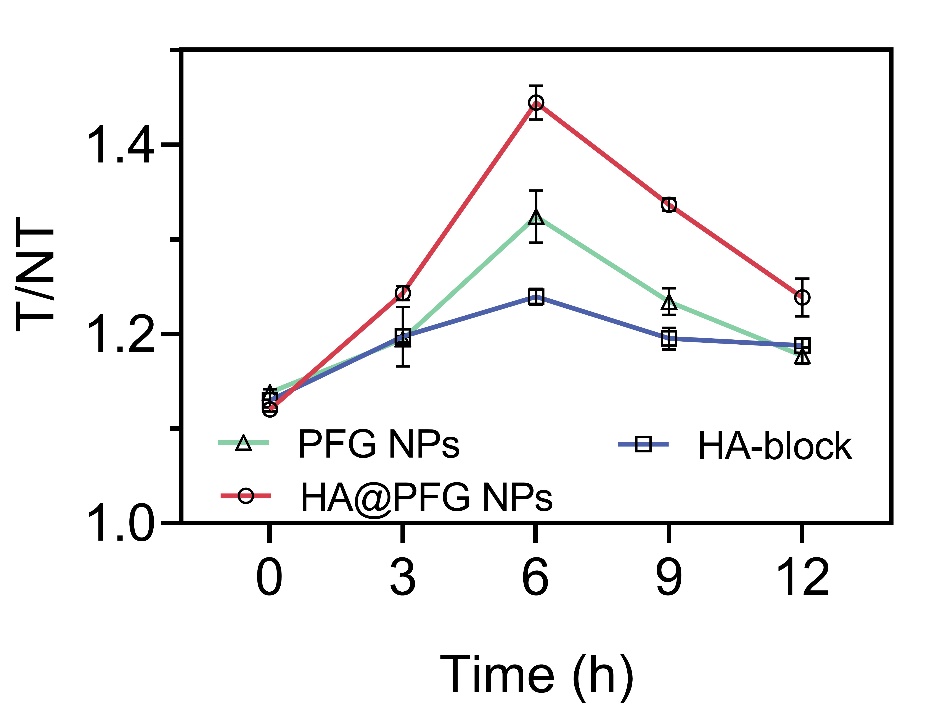


Figure S8. Relative intensity of the T1-weighted signal of the tumor site at different time points.


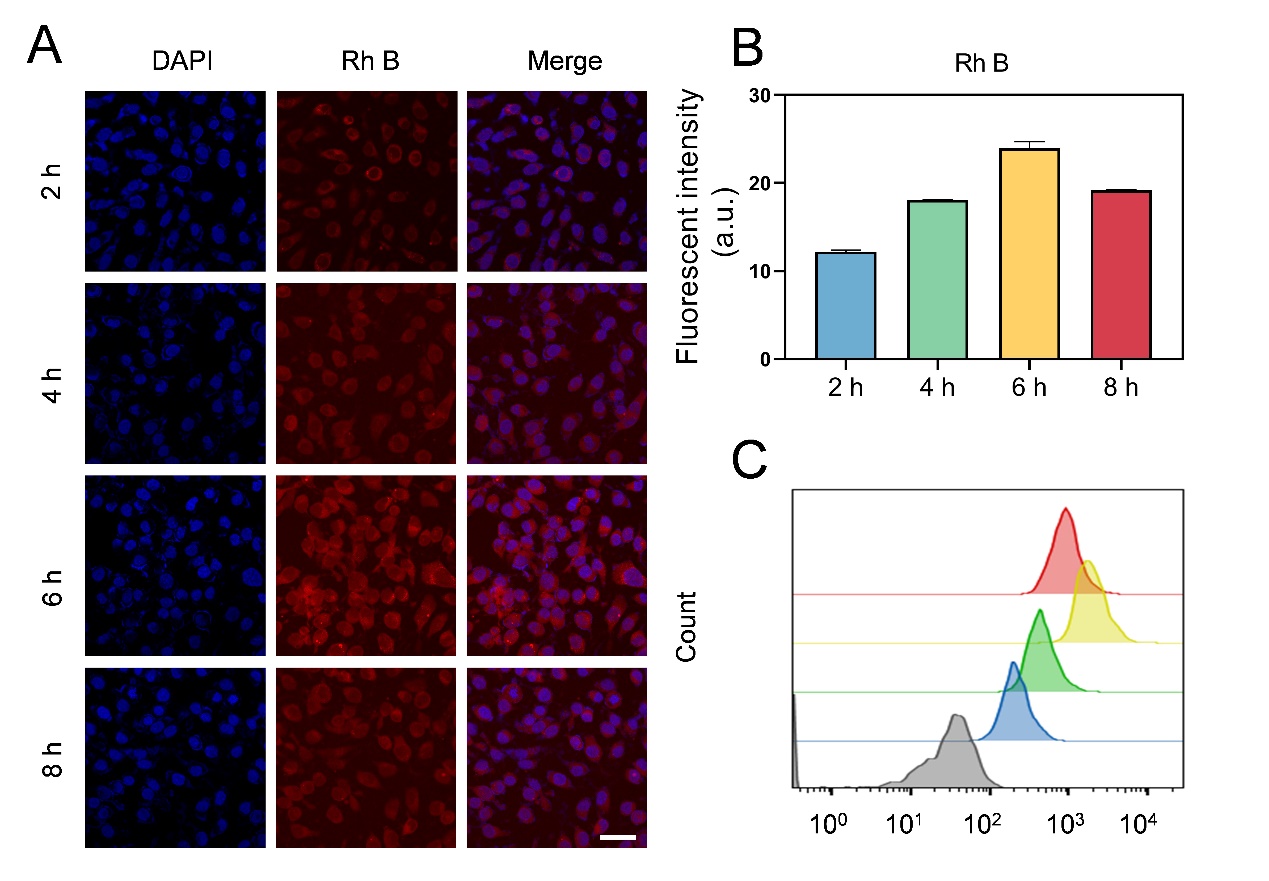


Figure S9. In vitro intracellular uptake at different time points. (A) CLSM of A2780 cells after incubation with HA@PFG NPs for 2, 4, 6, and 8 h. The cell nucleus was stained by DAPI, Scale bar = 25 μm. (B) Semi-quantitative analysis of mean Rho B intensity of intracellular HA@PFG NPs. (C) Quantitative analysis of intracellular NPs by flow cytometry.


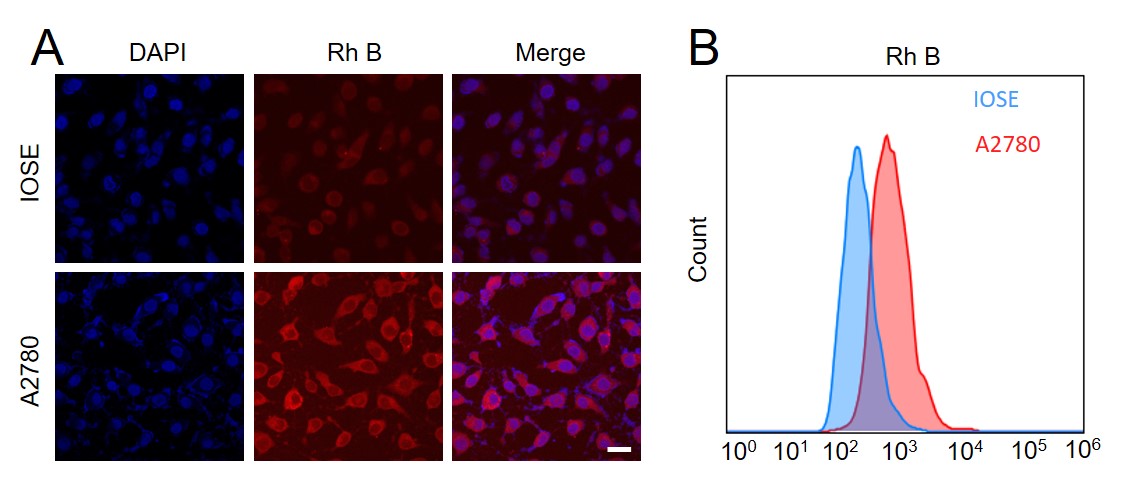


Figure S10. In vitro intracellular uptake of A2780 and IOSE cells. (A) CLSM of A2780 and IOSE cells after incubation with HA@PFG NPs for 6h. The cell nucleus was stained by DAPI, Scale bar = 25 μm. (B)Quantitative analysis of intracellular NPs by flow cytometry.


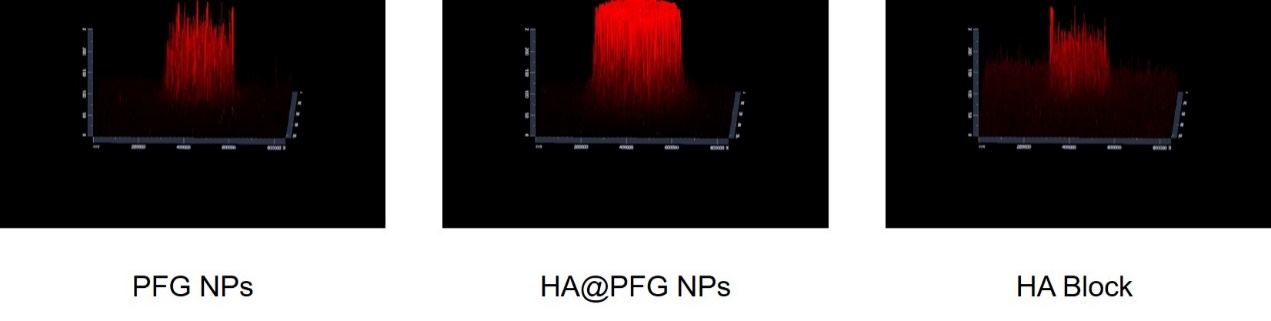


Figure S11. 2.5D images observed by CSLM show the relative drug intake of A2780 sphere.


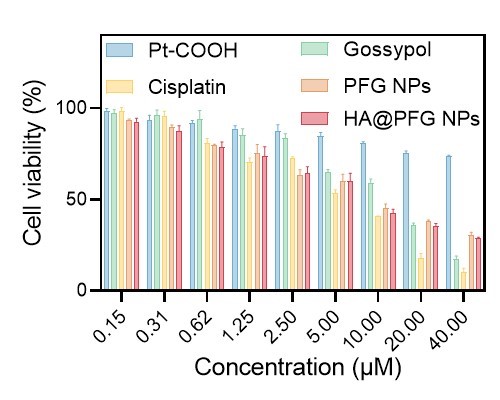


Figure S12 Relative cell viability of IOSE cells after treatment with Pt-COOH, Gossypol, Cisplatin, PFG NPs, and HA@PFG NPs for 72 h.


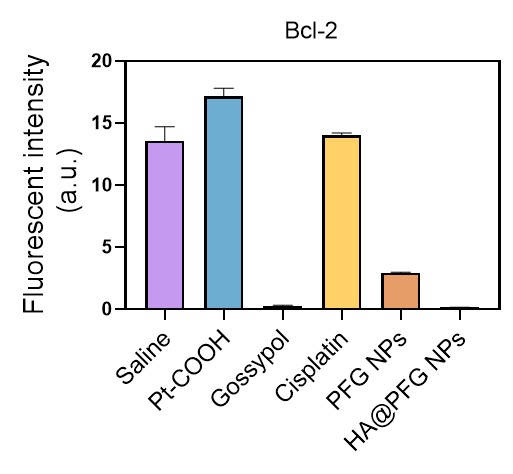


Figure S13. The semi-quantitation of Bcl-2 fluorescence intensity in different groups.


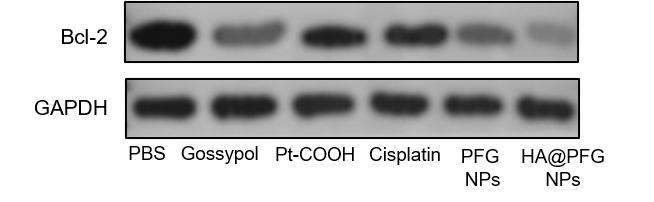


Figure S14. Western blot analysis of Bcl-2 proteins in tumor tissue after various treatments.

Figure S15. The semi-quantitation of GPX4 fluorescence intensity in different groups.


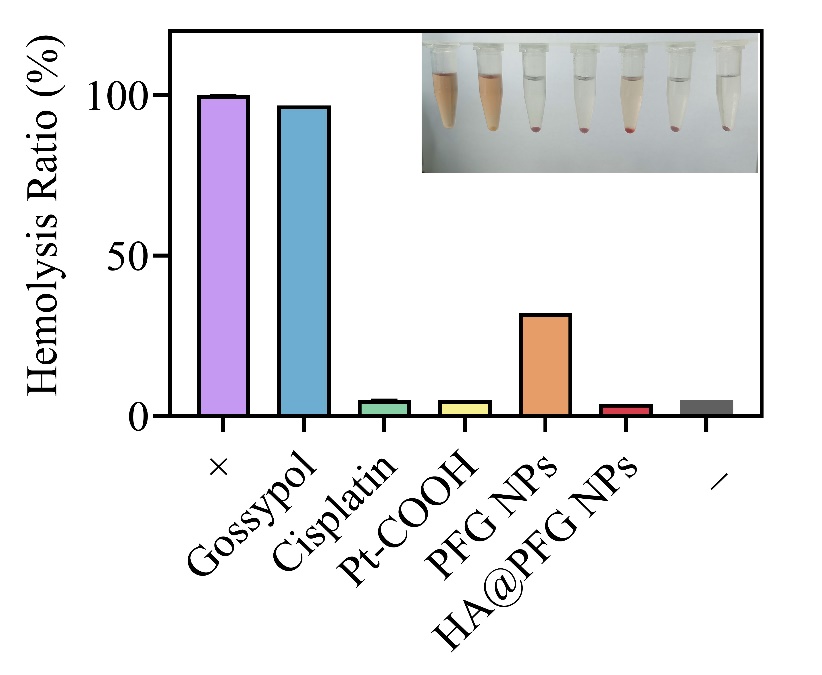


Figure S16. Hemolysis test result of gossypol, Cisplatin, Pt-COOH, PFG NPs and HA@PFG NPs.


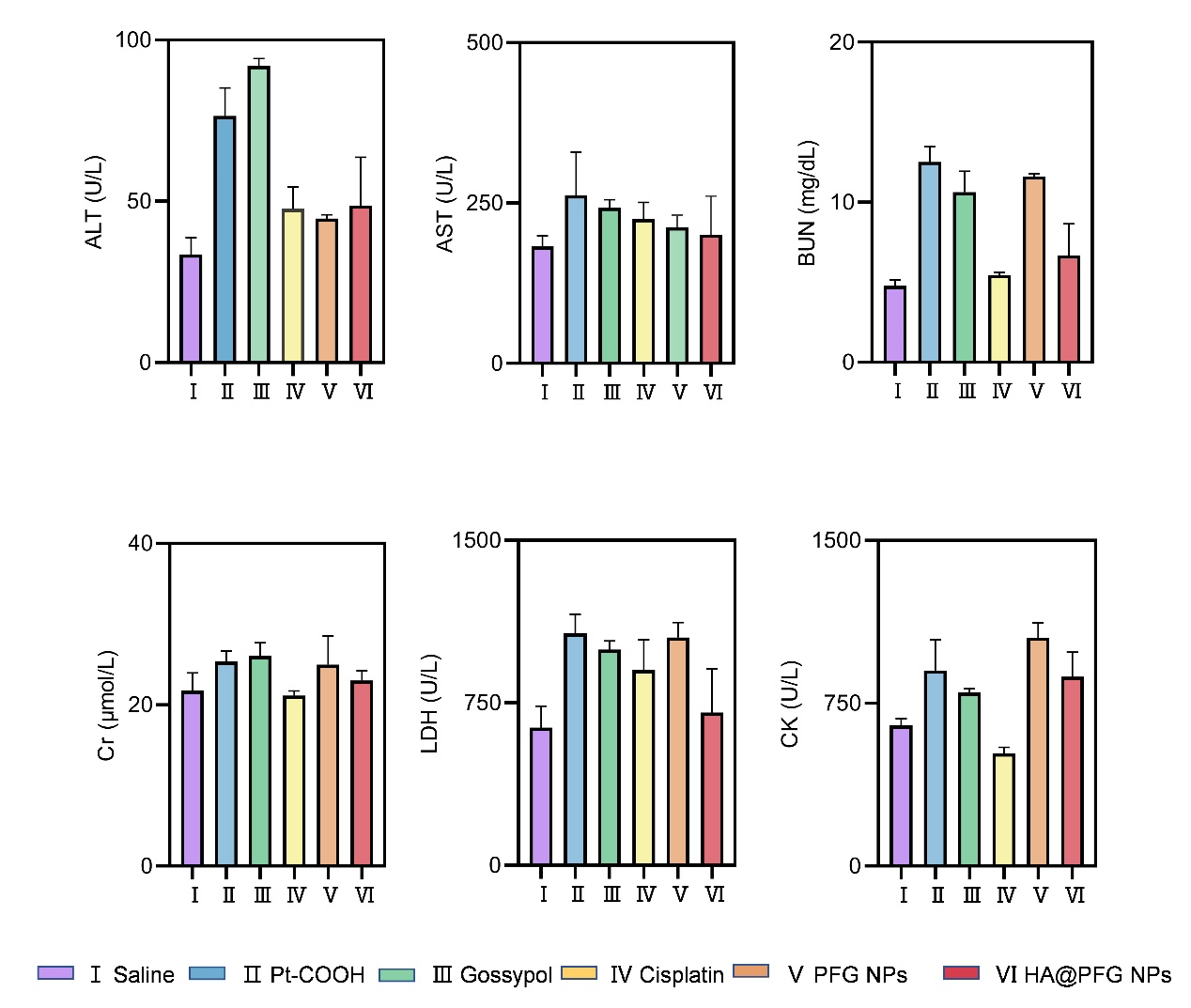


Figure S17. Levels of the main serum biochemical indicators of different groups after treatment (n=3).


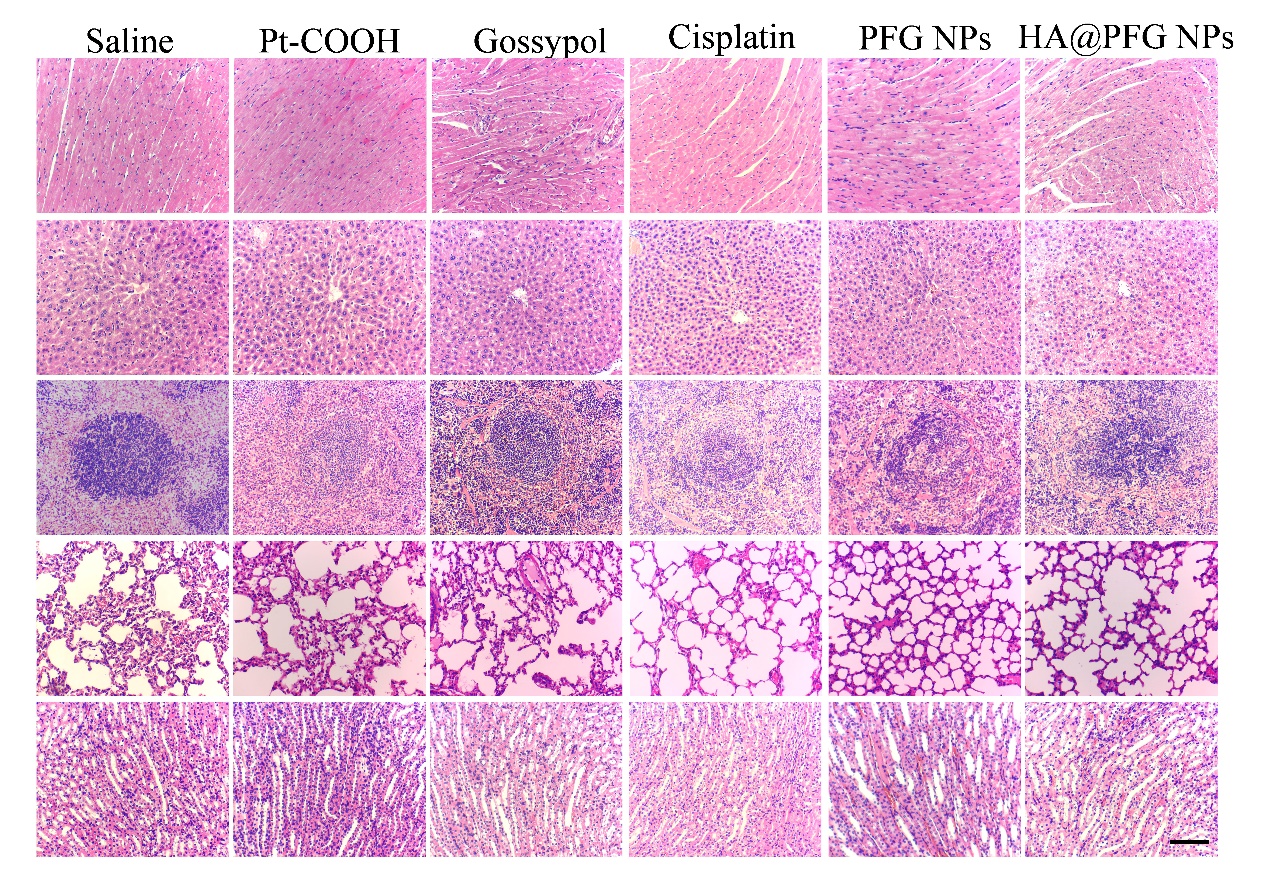


Figure S18. Representative H&E-stained histological images of main organs after treatment with different agents. Scale bar: 100 μm
